# Intrinsic Cross-Correlation Analysis of Hydro-Meteorological Data in the Loess Plateau, China

**DOI:** 10.3390/ijerph17072410

**Published:** 2020-04-02

**Authors:** Xiaowei Wei, Hongbo Zhang, Xinghui Gong, Xingchen Wei, Chiheng Dang, Tong Zhi

**Affiliations:** 1 School of Environmental Science and Engineering, Chang’an University, Xi’an 710054, China; 2017129014@chd.edu.cn (X.W.); gongxinghui@chd.edu.cn (X.G.); 2018129022@chd.edu.cn (X.W.); 2018129020@chd.edu.cn (C.D.); 2018129009@chd.edu.cn (T.Z.); 2 Key Laboratory of Subsurface Hydrology and Ecological Effect in Arid Region, Ministry of Education, Chang’an University, Xi’an 710054, China

**Keywords:** intrinsic correlation, detrended partial cross-correlation analysis, temporal evolution, scaling correlation, non-stationary data

## Abstract

The purpose of this study is to illustrate intrinsic correlations and their temporal evolution between hydro-meteorological elements by building three-element-composed system, including precipitation (P), runoff (R), air temperature (T), evaporation (pan evaporation, E), and sunshine duration (SD) in the Wuding River Basin (WRB) in Loess Plateau, China, and to provide regional experience to correlational research of global hydro-meteorological data. In analysis, detrended partial cross-correlation analysis (DPCCA) and temporal evolution of detrended partial-cross-correlation analysis (TDPCCA) were employed to demonstrate the intrinsic correlation, and detrended cross-correlation analysis (DCCA) coefficient was used as comparative method to serve for performance tests of DPCCA. In addition, a novel way was proposed to estimate the contribution of a variable to the change of correlation between other two variables, namely impact assessment of correlation change (IACC). The analysis results in the WRB indicated that (1) DPCCA can analyze the intrinsic correlations between two hydro-meteorological elements by removing potential influences of the relevant third one in a complex system, providing insights on interaction mechanisms among elements under changing environment; (2) the interaction among P, R, and E was most strong in all three-element-composed systems. In elements, there was an intrinsic and stable correlation between P and R, as well as E and T, not depending on time scales, while there were significant correlations on local time scales between other elements, i.e., P-E, R-E, P-T, P-SD, and E-SD, showing the correlation changed with time-scales; (3) TDPCCA drew and highlighted the intrinsic correlations at different time-scales and its dynamics characteristic between any two elements in the P-R-E system. The results of TDPCCA in the P-R-E system also demonstrate the nonstationary correlation and may give some experience for improving the data quality. When establishing a hydrological model, it is suitable to only use P, R, and E time series with significant intrinsic correlation for calibrating model. The IACC results showed that taking pan evaporation as the representation of climate change (barring P), the impacts of climate change on the non-stationary correlation of P and R was estimated quantitatively, illustrating the contribution of climate to the correlation variation was 30.9%, and that of underlying surface and direct human impact accounted for 69.1%.

## 1. Introduction

Complex hydro-meteorological systems contain various interactions among hydro-meteorological elements [1,2]. Investigating cross-correlations and teleconnections between hydro-meteorological signals benefits to deeply understanding the whole hydrological system and to serve for water resources management [3]. Under the influences of changing climate and anthropogenic disturbance [4], the single hydro-meteorological time series and their interaction relationships tend to exhibit complex [5,6], non-stationary [7], and multi-scale [8] changes, and simple correlation tests between two variables may fail in demonstrating their intrinsic correlations and its evolution, bringing new challenges to understanding hydro-meteorological system under changing environment.

Cross-correlation analysis is a critical approach to reveal the characteristics of complex hydrologic processes [9], and can serve for model building, data assimilation, and engineering design [1]. Many indexes of correlation have been proposed and applied in the fields of finance, biology, meteorology, and hydrology [10,11,12,13,14], such as commonly Person correlation coefficient [10,11], Kendall’s tau coefficient [12], Spearman’s rho coefficient [13], Gini’s gamma coefficient [14], and so on. However, it should be noted that different methods have application limitations. Therein, Person correlation coefficient can only reflect the degree of linear correlation between variables, and Kendall’s tau coefficient, Spearman’s rho coefficient, and Gini’s gamma coefficient are able to reflect the nonlinear correlation between variables, but insufficient to describe dependence structure and characteristics [12,13,14,15]. With the emerging of non-stationary problems, correlation analysis between non-stationary variables has been paid much attention, and scholars worldwide have consecutively put forward the detrended cross-correlation analysis (DCCA) cross-correlation coefficient [16,17], detrended partial cross-correlation analysis (DPCCA) [18], temporal evolution of detrended cross-correlation analysis (TDCCA) correlation coefficient, and temporal evolution of detrended partial-cross-correlation analysis (TDPCCA) [19], which have been applied in the fields of meteorology and hydrology [17,19,20].

The above-mentioned DCCA cross-correlation coefficient is defined based on the relevant parameters in detrended fluctuation analysis (DFA) and DCCA, aiming to quantify the level of cross-correlation between non-stationary time series and to identify seasonal components [16]. DPCCA is a DCCA-based improved method by including partial-correlation technique, which advantages are to handle non-stationary signals and reveal intrinsic correlations between two time series by removing potential influences of other unconsidered signals [18]. The intrinsic cross-correlations between variables are significant to exploring the complex changes and its non-stationarity evolution in the system. Shen et al. (2016) analyzed the intrinsic cross-correlations between the air pollution index (API) records and synchronously meteorological elements data through DPCCA, and found that the detrended partial cross-correlation coefficient between API and meteorological elements varied with spatial location and seasons [20]. Yan et al. (2017) employed the DPCCA to investigate horizontal oil-water two-phase flow structures, and found that DPCCA cross-correlation coefficient can serve as an effective indicator of horizontal oil-water two-phase flow structures [21]. Liu et al. (2017) investigated age-related changes of event-related potentials with DPCCA to explore the discrimination between the young and the older [22].

Afterward, the TDCCA and TDPCCA were successively proposed based on DCCA and DPCCA, by which it is feasible to explore the correlations on multi-time scales and at different periods. However, they are rarely introduced and applied into hydrological fields to study the temporal evolution of correlation between hydro-meteorological variables, though the cross-correlation analysis methods for non-stationary series have been developed rapidly in recent years. In addition, few scholars diagnosed the intrinsic correlation between hydro-meteorological elements.

In highly complex and irregular hydrological system/process, a seemingly random hydrological process is not a simple change, and could be the result of nonlinear interactions among a few dominant variables [23]. In the catchment hydrologic cycle in arid and semi-arid area, a small change of meteorological element could have a great influence on the hydrological process under the changing environment. For example, an increase/decrease as well as spatial changes in precipitation and other factors like evaporation may lead to a significant variation in discharge [24]. It is also well known that the change in evaporations is always dominated by precipitation change in arid and semi-arid area, and meanwhile is driven by temperature, wind speed, relative humidity, and other factors used in determining evaporation capacity [25]. Thus, it is very clear that different hydro-meteorological elements influence each other, and present time-varying nonstationary correlations among them in the system, where interactions are more difficult to understand and forecast.

In order to provide new insights into the interaction processes in complex hydrological system, intrinsic cross-correlation and its temporal evolution dynamic between hydro-meteorological elements, i.e., precipitation (P), evaporation (E), runoff (R), air temperature (T), and sunshine duration (SD) in Wuding River Basin (WRB) in the Loess Plateau were analyzed systematically in this study with DCCA cross-correlation coefficient, DPCCA, and TDPCCA, and the non-stationarities of the correlations were explored more intuitively, aiming to provide regional experience to correlational research between global hydro-meteorological variables and to further serve for improving the data quality used in building hydrologic model. In addition, a new way was proposed in the study to estimate the contribution of a related variable to the nonstationary change of correlation between other two variables in a system, namely impact assessment of correlation change (IACC), combining DCCA cross-correlation coefficient and DPCCA. In the case study of the WRB, the impacts of climate, underlying surface, and direct human activities to the nonstationary change of correlation between P and R, were evaluated through the IACC, with the purpose of providing some technical support for impact assessment of climate change in arid and semi-arid area.

## 2. Study Area and Data

**Figure 1:**
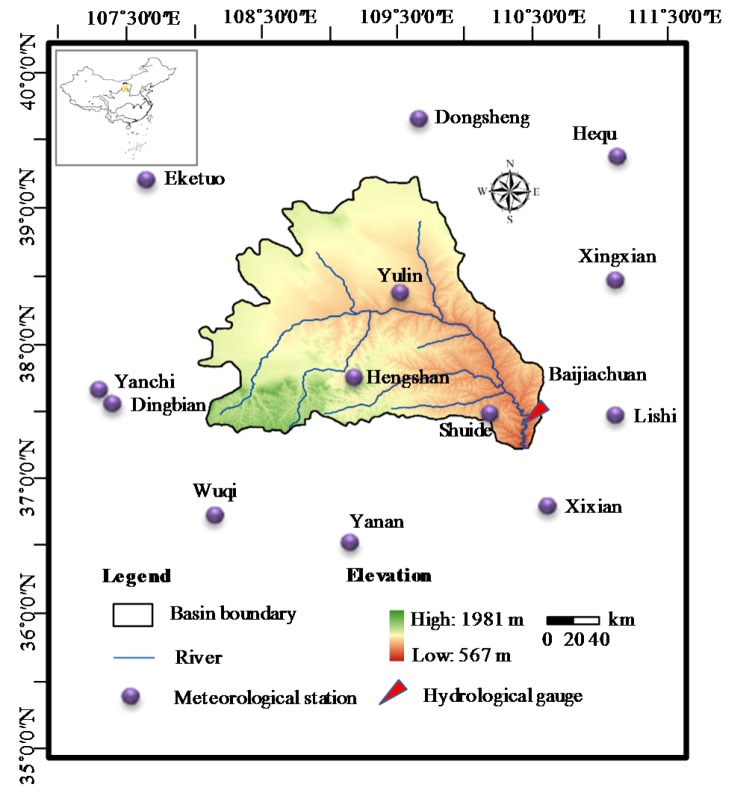
Locations of the study area, hydrological gauge, and meteorological stations.

The Wuding River is a first-order tributary of the Yellow River, originating from the Baiyu Mountain in Dingbian county and flowing into the Yellow River in Qingjian county of northern Shaanxi province, China. The Wuding River Basin (WRB) is located in a transition zone from farmland and grassland to desert over the Loess Plateau of northern China (Figure 1), influenced by a temperate and semi-arid continental monsoon climate [26]. The basin covers a catchment area of 30,261 km^2^, located at 37°14′–39°35′ N and 108°18′–111°45′ E [5]. Heavily influenced by the monsoon climate, the rainfall is quite unevenly distributed, causing rainstorms often to happen, but annual rainfall is very low. The average annual precipitation varies from 300 to 550 mm, and the average annual discharge is 15.3 × 10^8^ m^3^. The WRB as a representative basin in the Loess Plateau, is characterized by sparse vegetation, serious soil erosion, and the fragile and sensitive hydrological ecosystem, impacted by excessive reclamation of human beings. It is reported that approximately 45.65% of the basin is covered by fine loess in the south and eastern part, and 54.35% is the coarse sandy hilly area (Musu desert) [27].

**Figure 2:**
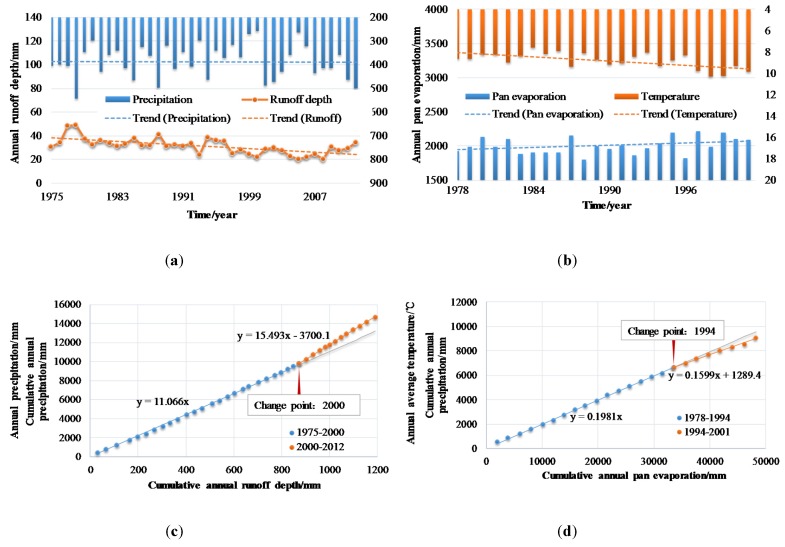
The variability of annual precipitation and runoff (**a**), pan evaporation and mean air temperature (**b**), double cumulative curves of annual precipitation and runoff depth (**c**), and annual precipitation and pan evaporation (**d**) in the Wuding River Basin (WRB).

In the past years, it is always regarded as a major basin in Loess Plateau to be harnessed, where the government carried out erosion and sediment control through water conservancy and soil conservation measures. However, influenced by climate change and these human disturbances, the measured hydro-meteorological data series in the WRB show non-stationary characteristics recently, as shown in Figure 2. It can be seen from the figure that annual runoff in the WRB shows a decreasing trend, while annual evaporation and temperature show a slightly rising trend. In addition, the double cumulative curves of P-R (Figure 2c) and P-E (Figure 2d) demonstrate obvious non-stationary changes occurring in the correlation between hydro-meteorological signals in the WRB, implying changes in the relationships of elements in the hydrology system. Hence, addressing the correlation and its change between the hydro-meteorological signals under the changing environment and understanding the interaction processes in the catchment system, may be of great significance to explore the hydrological response to climate change in the loess plateau.

The study selected the monthly runoff data at Baijiachuan hydrological gauge on the main Wuding river, and monthly meteorological data at 13 stations in or around the WRB. The locations of the hydrological gauge and meteorological stations are shown in Figure 1. Therein, the runoff data were obtained from hydrological manuals published by Hydrology Bureau of the Yellow River Conservancy Commission (YRCC), China. The meteorological data, including precipitation (P), pan evaporation (E), air temperature (T), and sunshine duration (SD), were provided by the National Climate Center of the China Meteorological Administration (http://data.cma.cn/). The Thiessen polygon method was adapted to convert point data into watershed scale. Due to the limitation of the length of available pan evaporation data, all time series used in this paper only cover the period of 1978–2001 year.

## 3. Methodology

### 3.1. DPCCA

The detrended partial cross-correlation analysis (DPCCA) method based on DCCA cross-correlation coefficient is proposed for quantifying the intrinsic correlation among non-stationary time series on different time scales by considering the possible interference of other relevant factors which may affect the results of correlation diagnosis and hydrological prediction [18]. In this study, the DPCCA method was mainly employed for testing the intrinsic correlation between two elements in three-element-coupled hydro-meteorological system by deducting the possible interference of the third element. The detailed calculation steps of DPCCA are as follows:

1. Suppose three time series $\left\{x_{t}^{1}\right\},$ $\{x_{t}^{2}\},$ $\{x_{t}^{3}\}$, where t=1,2,3,…,N. We can define cumulative series as
  (1) $$P_{k}^{j}\equiv \sum_{i=1}^{k}x_{t}^{j},$$
  where j=1,2,3, k=1,2,3,…,N.
2. Divide the entire cumulative series into N−s overlapping boxes, where s (n+1<s<N−1) represents the difference between sequence numbers corresponding to the first and last values in a box. Each box i (i=1,2,…,N−s) contains s+1, values, starting at i and ending at i+s where s+1 represents the time scale. In each box i, we can determine the ‘‘local trend’’ $P_{k,i}^{j}\;(i\leq k\leq i+s)$ by using a polynomial fit, and the linear fit was adopted in this study (n=1). Accordingly, the time scale s+1 will vary between n+3 and N−1. For climatic records, the second-order polynomial fit is normally enough. Then the detrended residual as the difference between the original cumulative series $P_{k}^{j}$ and the local trend $P_{k,i}^{j}$, namely “detrended walk”, can be further defined as
  (2) $$Y_{(i-1)(s+1)+k-i+1}^{j}=P_{k}^{j}-\tilde{P_{k,i}^{j}}$$
  where j=1,2,3, k=1,2,3,…,N, *n +* 1 < *s* < *N*−1.
3. Based on each detrended residual series $Y_{l}^{j},l=1,2,3,\ldots ,(N-s)(s+1)$ corresponding to $\left\{x_{i}^{j}\right\}$, the covariance between any two residuals can be calculated as
  (3) $$F_{j_{1}{,j}_{2}}^{2}(s)=\frac{\sum_{l=1}^{\left(N-s)(s+1\right)}Y_{l}^{j_{1}}Y_{l}^{j_{2}}}{\left(N-s)(s+1\right)},$$
  where $j_{1}{,j}_{2}=1,2,3$. Then, a covariance matrix can be constructed as
  (4) $$F^{2}(s)=\left(\begin{array}{c} F_{1,1}^{2}{(s),F}_{1,2}^{2}{(s),F}_{1,3}^{2}\left(s\right)\\ F_{2,1}^{2}{(s),F}_{2,2}^{2}{(s),F}_{2,3}^{2}\left(s\right)\\ F_{3,1}^{2}{(s),F}_{3,2}^{2}{(s),F}_{3,3}^{2}\left(s\right) \end{array}\right)$$
4. The cross-correlation levels between any two series, i.e., $\left\{x_{i}^{j_{1}}\right\}$ and $\left\{x_{i}^{j_{2}}\right\}$, can be estimated as
  (5) $$F^{2}(s)=\left(\begin{array}{c} F_{1,1}^{2}{(s),F}_{1,2}^{2}{(s),F}_{1,3}^{2}\left(s\right)\\ F_{2,1}^{2}{(s),F}_{2,2}^{2}{(s),F}_{2,3}^{2}\left(s\right)\\ F_{3,1}^{2}{(s),F}_{3,2}^{2}{(s),F}_{3,3}^{2}\left(s\right) \end{array}\right)$$
  and the coefficients matrix can further be obtained as
  (6) $$\rho (s)=\left(\begin{array}{c} \rho_{1,1}{(s),\rho }_{1,2}{(s),\rho }_{1,3}\left(s\right)\\ \rho_{2,1}{(s),\rho }_{2,2}{(s),\rho }_{2,3}\left(s\right)\\ \rho_{3,1}{(s),\rho }_{3,2}{(s),\rho }_{3,3}\left(s\right) \end{array}\right),$$
  where $-1\leq \rho_{j_{1}{,j}_{2}}\left(s\right)\leq 1$, represents the level of cross-correlation on time scales of s+1. It is the so-called DCCA cross-correlation coefficients.

However, it should be noted that it only shows the relations between the series $\left\{x_{i}^{j_{1}}\right\}$ and $\left\{x_{i}^{j_{2}}\right\}$. The correlation may provide spurious information if the two series are both correlated with other signals [19]. Therefore, it is essential to combine the partial-correlation technique to eliminate the potential influence of other variable.

5. Before using the partial-correlation technique, the inverse matrix of ρ(s) can be calculated as
  (7) $${C(s)=\rho }^{-1}(s)=\left(\begin{array}{c} C_{1,1}{(s),C}_{1,2}{(s),C}_{1,3}\left(s\right)\\ C_{2,1}{(s),C}_{2,2}{(s),C}_{2,3}\left(s\right)\\ C_{3,1}{(s),C}_{3,2}{(s),C}_{3,3}\left(s\right) \end{array}\right)$$
6. For any two series $\left\{x_{i}^{j_{1}}\right\}$ and $\left\{x_{i}^{j_{2}}\right\}$, the partial-cross-correlation level can be determined as
  (8) $$\rho_{\mathrm{DPCCA}}{(j}_{1}{,j}_{2};s)=\frac{{-C}_{j_{1}{,j}_{2}}\left(s\right)}{\sqrt{C_{j_{1}{,j}_{1}}\left(s\right)\cdot C_{j_{2}{,j}_{2}}\left(s\right)}},$$
  where the coefficients $\rho_{\mathrm{DPCCA}}{(j}_{1}{,j}_{2};s)$ can be used to characterize the “intrinsic” correlation between the two series on time scales of s+1. Then, the partial cross-correlation levels on different time scales can be further estimated by changing *s*. Similar to Pearson correlation coefficient, the larger the absolute value of the coefficients $\rho_{\mathrm{DPCCA}}{(j}_{1}{,j}_{2};s)$ is, the stronger the correlation is. In other words, the closer the coefficients $\rho_{\mathrm{DPCCA}}{(j}_{1}{,j}_{2};s)$ are to 1 or −1, the stronger the correlation is, while the closer $\rho_{\mathrm{DPCCA}}{(j}_{1}{,j}_{2};s)$ is to 0, the weaker the correlation is. In addition, the degree of correlation can be determined through the range of correlation coefficient, including extremely strong, strong, medium, weak, and extremely weak/no, as shown in Table 1.

### 3.2. TDPCCA

Temporal evolution of detrended partial-cross-correlation analysis (TDPCCA) is the development of DPCCA, proposed to detect the multi-time scales correlation with temporal evolution and for multi-variables [19]. In this study, the TDPCCA method is also mainly used for three-element-coupled hydro-meteorological system. Specific calculation steps are as follows:

1. For a given time scale s+1, based on the results in step 1 and 2 in Section 3.1, the detrended residual sequences can built the point-to-points structure as follows
  (9) $$\{\begin{array}{c} Y_{1,k}^{j}{=Y}_{t_{1}}^{j}\to t_{1}=1+(s-1)/2\\ Y_{2,k}^{j}{=Y}_{t_{2}}^{j}\to t_{2}=2+(s-1)/2\\ \ldots \\ Y_{i,k}^{j}{=Y}_{i}^{j}\to t_{i}=i+(s-1)/2 \end{array}$$
2. Then a new matrix can be obtained for the time series $\left\{x_{i}^{j}\right\}$ as
  (10) $$\left(\begin{array}{c} Y_{t_{1}}^{1}Y_{t_{2}}^{1}\cdots Y_{t_{i}}^{1}\\ Y_{t_{1}}^{2}Y_{t_{1}}^{2}\cdots Y_{t_{i}}^{2}\\ Y_{t_{1}}^{3}Y_{t_{2}}^{3}\cdots Y_{t_{i}}^{3} \end{array}\right)$$
3. According to Equations (3) and (5), the cross-correlation coefficients between two series $\left\{x_{i}^{j_{1}}\right\}$ and $\left\{x_{i}^{j_{2}}\right\}$ can be calculated, as follows
  (11) $$\rho (j_{1},j_{2};s;t)=[\rho_{t_{1}}^{j_{1}{}_{,}j_{2}}(s),\rho_{t_{2}}^{j_{1}{}_{,}j_{2}}(s),\cdots ,\rho_{t_{i}}^{j_{1}{}_{,}j_{2}}(s)]$$
4. For time point $t_{i}$, the correlation matrix by using Equations (3) and (5) can be obtained.
  (12) $$\rho \left({s;t}_{i}\right)=\left(\begin{array}{ccc} 1 & \rho_{t_{i}}^{1,2}\left(s\right) & \rho_{t_{i}}^{1,3}\left(s\right)\\ \rho_{t_{i}}^{2,1}\left(s\right) & 1 & \rho_{t_{i}}^{2,3}\left(s\right)\\ \rho_{t_{i}}^{3,1}\left(s\right) & \rho_{t_{i}}^{3,2}\left(s\right) & 1 \end{array}\right)$$
5. According to Equation (7), the inverse matrix of $\rho \left({s;t}_{i}\right)$ can be calculated.
  (13) $$C\left(s;t_{i}\right)=\rho^{-1}\left(s;t_{i}\right)=(\begin{array}{cccc} C_{t_{i}}^{1,1}\left(s\right) & C_{t_{i}}^{1,2}\left(s\right) & \cdots & C_{t_{i}}^{1,m}\left(s\right)\\ C_{t_{i}}^{2,1}\left(s\right) & C_{t_{i}}^{2,2}\left(s\right) & \cdots & C_{t_{i}}^{2,m}\left(s\right)\\ \vdots & \vdots & & \vdots \\ C_{t_{i}}^{m,1}\left(s\right) & C_{t_{i}}^{m,2}\left(s\right) & \cdots & C_{t_{i}}^{m,m}\left(s\right) \end{array})$$
6. The partial-cross-correlation coefficients can be estimated by the following equation for any two series $\left\{x_{i}^{j_{1}}\right\}$ and $\left\{x_{i}^{j_{2}}\right\}$.
  (14) $$\rho_{\mathrm{TDPCCA}}{(j}_{1}{,j}_{2}{;s;t}_{i})=\frac{{-C}_{t_{i}}^{j_{1}{,j}_{2}}\left(s\right)}{\sqrt{C_{t_{i}}^{j_{1}{,j}_{1}}(s)\;\cdot {\;C}_{t_{i}}^{j_{2}{,j}_{2}}\left(s\right)}}$$

### 3.3. Impact Assessment of Correlation Change

For a non-stationary system, it is very significant that understanding the correlation between two correlated elements and quantifying the non-stationary change of correlation induced by other elements, due to it can contribute to exploring the non-stationary structure of the system. In this work, a novel mathematical way was proposed for estimating the contribution of one element in a system to the change of correlation between two other series, namely impact assessment of correlation change (IACC), which was developed by combining DCCA cross-correlation coefficient, DPCCA, TDCCA and TDPCCA.

In Section 3.2, the correlation coefficient $\rho_{t_{i}}^{j_{1}{,j}_{2}}\left(s\right)$ and partial correlation coefficient $\rho_{\mathrm{TDPCCA}}{(j}_{1}{,j}_{2}{;s;\;t}_{i})$ at time $t_{i}$ on time scale s+1 have been calculated. By developing the two correlation coefficients, the impacts or contributions of one factor to the change of correlation between the other two factors can be roughly defined as coefficient *C*, and the calculation model is called as the IACC-1 model. The detail is described as follows

(15) $$\;C\left(j_{1}{,j}_{2}{;\;j}_{3}{;\;t}_{i}\right)=\left|\frac{\rho_{\mathrm{TDPCCA}}\left(j_{1}{,j}_{2}{;s;t}_{i}\right)-\rho_{t_{i}}^{j_{1}{,j}_{2}}\left(s\right)}{{1-\rho }_{t_{i}}^{j_{1}{,j}_{2}}\left(s\right)}\right|$$

Analogously, combining cross-correlation coefficients of DCCA and DPCCA, the average level of the contribution of one factor to the change of correlation between other two factors can be estimated, and the corresponding model is named as the IACC-2. The formula is as follows

(16) $$\;C\left(j_{1}{,j}_{2}{;\;j}_{3}\right)=\frac{1}{N-n-3}\overset{N-2}{\underset{s=n+2}{\sum }}\left|\frac{\rho_{DPCCA}\left(j_{1}{,j}_{2};\;s\right)-\rho_{j_{1}{,j}_{2}}\left(s\right)}{{1-\rho }_{j_{1}{,j}_{2}}\left(s\right)}\right|$$

It is worth noting that IACC-1 model illustrates the temporal evolution of impact of one factor to the change of investigative correlation on certain time scale, while IACC-2 model demonstrate the average impact of that over the whole time span.

## 4. Results

### 4.1. Correlations of Hydro-Meteorological Variables

The correlations among hydro-meteorological variables (i.e., P, E, R, T, and SD) in the WRB were calculated by the DPCCA, and were compared with that by the DCCA cross-correlation coefficient. In terms of three-element-composed system, some new correlation-based insights on hydro-meteorological system interaction mechanisms were obtained.

#### 4.1.1. P-R-E

**Figure 3:**
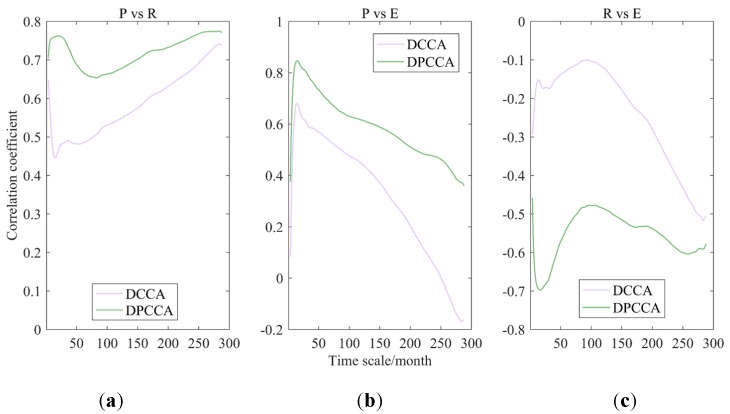
Cross-correlation coefficients among precipitation (P), runoff (R), and evaporation (E) obtained by detrended cross-correlation analysis (DCCA) cross-correlation coefficient and detrended partial cross-correlation analysis (DPCCA), i.e., P-R (**a**), P-E (**b**), and R-E (**c**).

Figure 3 shows the results of correlation coefficients between P, R, and E through DCCA cross-correlation coefficient and DPCCA. It can be seen by introducing partial correlation that the absolute values of correlation coefficients between any two factors in P-R-E system increased to different degree with multiple time scales, and correlations among three variables showed better. Meanwhile, the variation of result differences between the DCCA coefficient and DPCCA coefficient reflected that the influence of the third factor on the relationship between two factors varied with time scales. To conclude, there are strong interactions among P, R, and E in the WRB. In addition, it is found from observed data that the correlation between P and R shows non-stationary tendency under the influence of warmer climate and unpredictable human activities, which may be contributed by the variation of E (or other coupled factors).

#### 4.1.2. P-E-T

**Figure 4:**
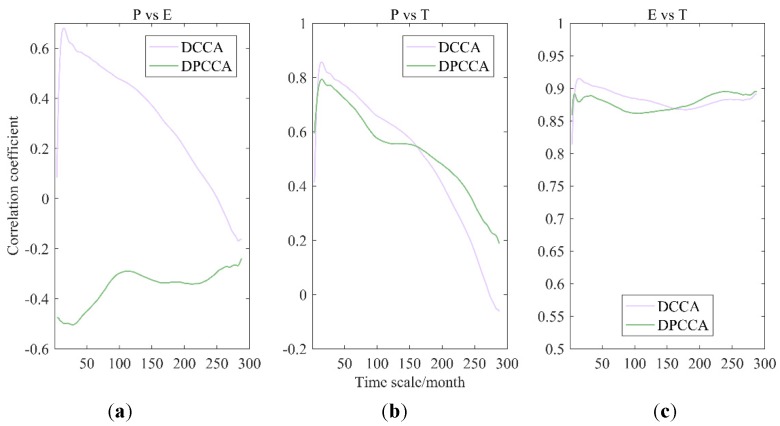
Cross-correlation coefficients among P, E, and air temperature (T) obtained by DCCA cross-correlation coefficient and DPCCA, i.e., P-E (**a**), P-T (**b**), and E-T (**c**).

As represented in Figure 4, with the influence of E (P) removed, changes of correlation coefficients between P and T (E and T) were very small, indicating that T is directly related to changes in regional precipitation or evaporation, and the third variable hardly affects the correlation of T and the other variable. However, when the interference of T was eliminated, cross-correlation coefficients between P and E changed greatly, revealing that T has a great influence on the correlation between P and E in the WRB. Obviously, P and E display a better correlation when the influence of T do not removed, so T should not be ignored when building the relationship between precipitation and evaporation. To evaluate the impact of T, significant test of intrinsic correlation will be further performed between P and T, and E and T in the following sections.

#### 4.1.3. P-E-SD

**Figure 5:**
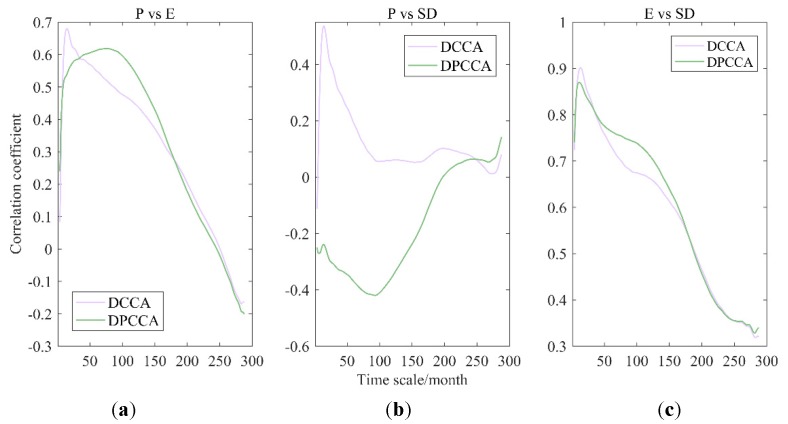
Cross-correlation coefficients among P, E, and sunshine duration (SD) obtained by DCCA cross-correlation coefficient and DPCCA, i.e., P-E (**a**), P-SD (**b**), and E-SD (c).

Cross-correlation coefficients among P, E, and SD by DCCA cross-correlation coefficient and DPCCA are presented in Figure 5. Compared to the analysis on three-element-composed system of P-R-E, the relationships among the three elements of P, E, and SD are difficult to identify directly and to give a clear physically-based driving mechanism. However, it can be observed that through the cross-correlation coefficients calculated in Figure 5, with the influence of SD removed, correlation coefficients between P and E changed slightly, implying that there was a direct and intrinsic correlation between P and E in small time-scales, not affected by SD. Similarly, the correlation between E and SD was also independent of P. Differently, P and SD showed extremely weak correlation at all time-scales when the interference of E is eliminated, implying that the system appears a correlation between P and SD depending E, but there is no direct relationship between them. It is consistent with people’s common understanding.

### 4.2. Testing for Significance of Intrinsic Correlation

**Figure 6:**
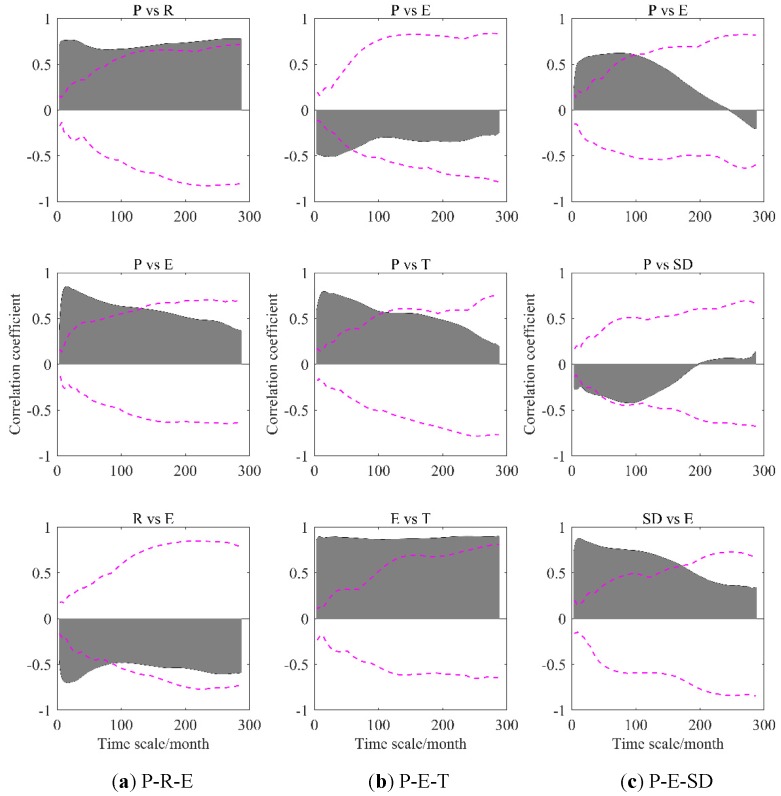
Significant tests of partial cross-correlation coefficients in a three-element-composed system, i.e., P-R-E (**a**), P-E-T (**b**), and P-E-SD (**c**). The upper (or lower) envelope of the black area is DPCCA coefficients. The dotted line notes the extreme values calculated by Monte-Carlo tests (100 times).

Although the correlations among hydro-meteorological variables at different time scales were discussed in the section above, the quantitative analysis has been not covered. In this part, significance tests of the DPCCA coefficients are conducted by Monte-Carlo simulation, aiming to provide some insights in the intrinsic significant correlation between hydro-meteorological variables at specific time-scales. Figure 6 shows the significant test results of intrinsic correlations in three-element-composed systems, i.e., P-R-E (a), P-E-T (b), and P-E-SD (c).

Specific time-scales with significant correlation, local peak value of correlation coefficients, time scale corresponding to peak value of correlation coefficients, and mean coefficient with significant correlation are reported in Table 2. Overall, it can be observed that time-scale-independent significant correlations between P and R represented in the P-R-E system, and that between E and T appeared in the P-E-T system. In P-E-T system, E and T were indeed positively correlated with mean correlation coefficient equal to 0.878, showing extremely strong correlation. While P and E and P and T were significantly correlated on time scales of less than 57 months and 109 months, respectively. This is approximately in agreement with previous study [28]. It is worth mentioning that the correlation between P and E varied with influence of the third variable (i.e., R or SD) removed. This result implies that the relationship between hydro-meteorological variables is complex and susceptible to other related variables.

### 4.3. Temporal Evolution of DPCCA

As mentioned above, DPCCA can effectively diagnose the intrinsic correlation between variables on multi-time scales with or without the influence of interference factors. However, what it reveals is an average level of correlation in time domain, and fails in reflecting temporal evolution details of correlation. Thus, the TDPCCA is employed to show correlations on multi-time scales and in different periods, aiming to explore the dynamics of correlation among hydro-meteorological variables. Next, as a case study, the temporal evolution of the correlations among P, R, and E in the WRB will be discussed detailedly through TDPCCA.

**Figure 7:**
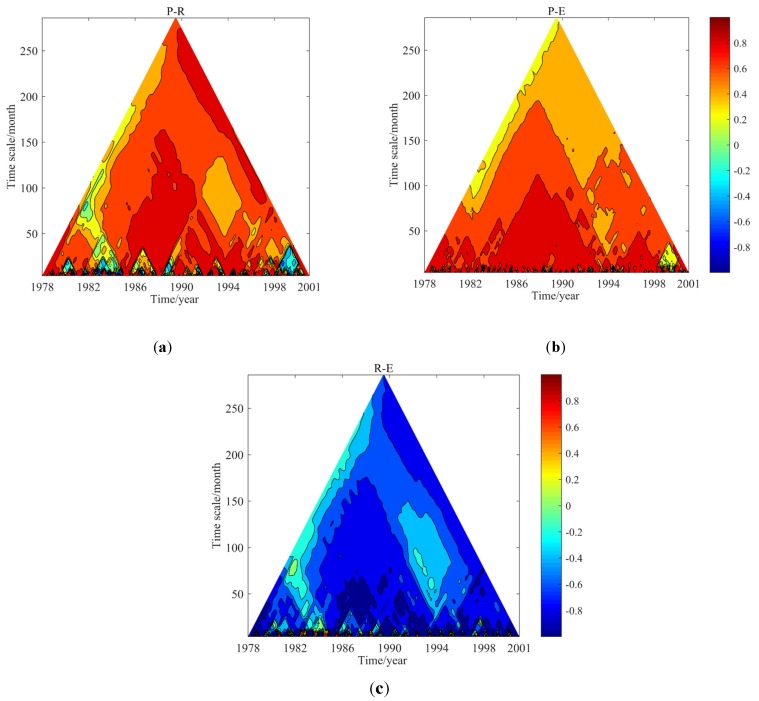
Temporal evolution of cross-correlation coefficients among P, R, and E obtained by temporal evolution of detrended partial-cross-correlation analysis (TDPCCA) i.e., P-R (**a**), P-E (**b**), and R-E (**c**).

Figure 7 displays the dynamics of correlation at different time-scales among P, R, and E. In Table 2, it is found that the P and R were time-scale-independent significant correlated in P-R-E system, however their relationship was not stationary. After removing the influence of E, it can be seen from correlation details in Figure 7a that P and R were correlated with very strong correlation at (or in) almost all time-scales and periods, representing more than 80% area in the figure with over 0.6, except 1978–1980 at 50–250-month scale, 1995–2001 at 50–140-month scale and the whole period at 4–50-month scale. It illustrates that the rainfall-runoff hydrological model built would have better performance if the precipitation and runoff data series in the period with strong correlation were used, implying the advantage of the correlation analysis on improving the data quality for hydrological model building. Similarly, Figure 7c indicates that R and E were correlated negatively with greatly strong correlation at almost all time-scales and periods except for the indigo area in the figure which was similar with non-significant areas in Figure 7a. Figure 7b depicts the evolution of correlations between P and E on different time-scales, and the areas with strong correlation were obviously less than the above two figures. In terms of the degree of correlation, P and E were generally correlated with highly strong correlation on time scales of less than about 10 years. Thus, it is clear that TDPCCA as a novel way, can effectively reveal the dynamics of correlation and time-scale-dependence in three-element-composed system, i.e., P-R-E, describing the temporal nonstationary relationship, and further provide valuable information for data selecting in hydrological model building.

## 5. Discussion

In Section 4, through DPCCA and DCCA cross-correlation coefficient, intrinsic correlations between hydro-meteorological data were analyzed and tested for statistical significance. Then, TDPCCA were applied in hydro-meteorological systems to investigate the temporal variation of correlation among P, R, and E, revealing nonstationary time-scale-dependent relationship. However, it is not enough since the causes of non-stationary correlation and quantitative analysis of the impacts of climate change and human activities were not considered. Therefore, the IACC-1 and IACC-2 model proposed in Section 3.3 were tentatively applied in this section to make the influence quantitative. Before that, the research works of this field needs to be summarized.

As well known, the interactions between climate and human systems are quite complicated and difficult to segregate [29,30]. In the late 1970s, global natural system variations as El Nino-Southern Oscillation (ENSO), tropical Atlantic SSTs, and Asian monsoon, play an important role in the earth drying. At local scale, climate change affects runoff production and impacts anthropogenic water diversion and governmental water resources management strategies. Moreover, human activities alter land use and land cover, and natural water resource allocation [31,32] and further influence the local even global hydrological cycle. In order to understand accurately the correlation and interaction mechanisms among hydrological variables in the changing environment, and to develop scientific strategies to cope with the changing climate, it is necessary to separate and quantify the contributions of climate change and human activities to hydrological variations (e.g., runoff and drought) [33,34,35] and even ecology elements (e.g., ecosystem evapotranspiration ET, gross primary productivity GPP) [29]. At present, the common models used for evaluating the impacts include Soil Water Assessment Tool (SWAT) model [31], Yellow river water balance model (YRWBM) model [33], Budyko-type equations [34], Penmann-Monteith model [35], and statistical analysis methods [29,36,37] mainly include integration of regression analysis and the double cumulative curve. Recently, statistical analysis methods are paid more attention due to they need less data and parameter calibration compared with hydrological models, and suitable to the data-scare watershed. However, it can be concluded from the relevant literatures that the main idea of statistical analysis method is to segment the sequence in order to choose a base period and then to estimate contributions by changing a certain driving element. Unfortunately, they often ignore mutual influences between variables in this process and cause the inaccurate estimations.

**Figure 8:**
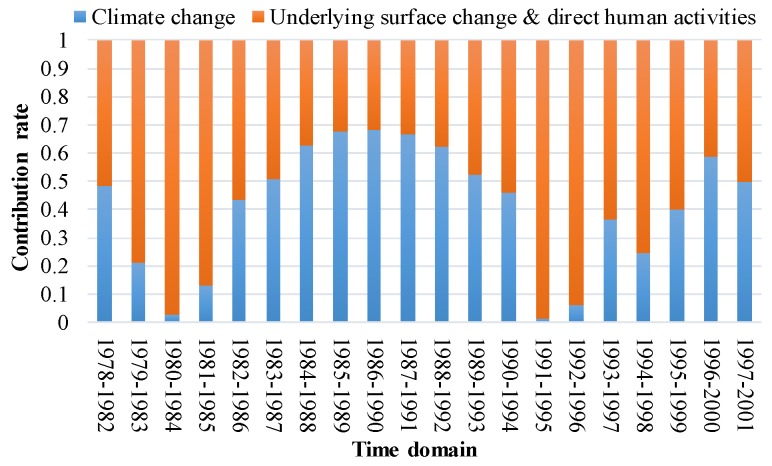
Contributions of climate change and underlying surface change and direct human activities to the correlation between P and R.

In this work, the impacts of climate change on the change of correlation between P and R in the WRB were evaluated, where the pan evaporation was employed as an indicator of climate variations representing integrated changes of temperature, wind speed, relative humidity, sunshine duration, and so on, i.e., climate impact except for P. Other effects on the non-stationary correlation between P and R was considered from human activities, including changes of the underlying surface and direct water diversion. The results obtained from IACC-2 combining DCCA cross-correlation coefficient and DPCCA show that the contribution of climate change to the non-stationary relationship between P and R in the WRB is 30.9%. Accordingly, the contribution of human activities is 69.1%. In terms of the principles of the IACC-2 model, it is clear that the results only provide a contribution rate over the whole period, indicating the influence of climate change or human activities to the change of correlation. Differently, IACC-1 model combining TDCCA and TDPCCA, can give the contributions changing over different periods, and the contributions of climate change and human activities to the change of correlation between P and R in the WRB are shown in Figure 8. The graph depicts their contributions on 5-year time scales.

In reality, the WRB has experienced a series of human-induced influences in the past time, such as reservoir construction and soil and water conservation (warping dam, returning farmland to forest, etc.), which caused great changes in the hydrologic cycle [4]. It can be seen from Figure 8 that the underlying surface and direct human activities played a dominant role in the change of correlation between P and R over two periods of 1980–1984 and 1991–1996, and the influence of climate change gradually increased after 1991. Relevant literature shows that a large number of small-sized check dams had almost been filled up in the WRB, and the siltation volume of large- and medium-sized check dams had reached 86% of the storage around 1990 [38]. The current situation of check dams explains the reason why the contribution of climate change to the correlation between P and R increases gradually after 1991.

## 6. Conclusions

In this study, intrinsic correlations and its dynamics among hydro-meteorological elements in the WRB in Loess Plateau, China, i.e., precipitation (P), runoff (R), air temperature (T), pan evaporation (E), and sunshine duration (SD), were analyzed with DPCCA and TDPCCA, compared with the correlations by DCCA cross-correlation coefficient. In addition, two novel formulas were proposed to estimate contributions of one element in three-element-composed system to the change of correlation between the other two elements, combining DPCCA and DCCA coefficient. Through the evaluation, the contributions of climate and human activities to the non-stationary correlations between P and R were estimated.

The results of intrinsic correlation analysis show that (1) some insights on the interaction mechanisms among hydro-meteorological variables (i.e., P, E, R, T, and SD) in the WRB are obtained. In all three-element-composed systems (i.e., P-R-E, P-E-SD, P-E-T), P-R-E system have the strongest interactions. In P-E-T system, T is directly related to changes in regional precipitation or evaporation, while P is indirectly correlated with SD via E in P-E-SD system. (2) There are stable significant correlations between P and R and E and T, not depending on time scales. However, there are significant correlations between other variables in P-R-E system and other systems on local time scales, such as P and E, R and E, P and T, P and SD, and E and SD, which present a time-scale-dependent correlation. (3) TDPCCA has advantage in revealing the intrinsic correlations at different time-scales and the dynamics characteristic of correlations between any two variables in P-R-E system. The analysis results in the P-R-E system also demonstrate that it can be used to describe the temporal nonstationary relationship, and further to provide valuable information for data selection in hydrological model building.

Impact assessment of correlation change (IACC) indicates that (1) IACC-1 and -2 models can estimate quantitatively the contribution of climate change (represented by pan evaporation, barring precipitation) on change of correlation between P and R. (2) The contribution of climate change to the change of correlation between P and R is 30.9%, and that of underlying surface and direct human activities is 69.1%.

## Figures and Tables

**Table 1:** Degree of correlation and the corresponding range of correlation coefficients.

Correlation Degree	Extremely Strong	Strong	Medium	Weak	Extremely Weak/No
Range of correlation coefficient	0.8–1.0	0.6–0.8	0.4–0.6	0.2–0.4	0–0.2

**Table 2:** Test result of significant correlation time-scales.

Three-Element-Composed System	P-R-E			P-E-T			P-E-SD		
	P-R	P-E	R-E	P-E	P-T	E-T	P-E	P-SD	E-SD
Significant time-scales (month)	Ind *	≤129	≤83	≤57	≤109	Ind	≤98	≤43	≤170
Peak value	0.762	0.847	−0.698	−0.504	0.793	0.891	0.618	−0.334	0.87
Time scale corresponding to peak value (month)	22	15	17	29	15	8	76	43	11
Mean coefficient	0.718	0.698	−0.595	−0.481	0.688	0.878	0.584	−0.29	0.739

^*^ Ind notes independent.
